# Beyond the Blot: Diagnostic Challenges in Atypical Dermatomyositis With Overlapping Myasthenia Gravis

**DOI:** 10.7759/cureus.92072

**Published:** 2025-09-11

**Authors:** Syed Mashood Iqbal, Veena Jayaramu, Sankar Ram Ragasankar, Sadiya Khan, Vivek Dubey

**Affiliations:** 1 Acute Medicine, Queen Elizabeth Hospital Birmingham, Birmingham, GBR; 2 Acute Medicine, Walsall Healthcare NHS Trust, Walsall, GBR; 3 Acute Medicine, Good Hope Hospital, Birmingham, GBR; 4 Acute Medicine, Aster CMI Hospital, Bangalore, IND

**Keywords:** acetylcholine receptor, autoimmune myopathy, dermatomyositis, dysphagia', muscle biopsy, myasthenia gravis (mg), myositis blot, overlap syndrome (os), pyridostigmine, seronegative

## Abstract

Dermatomyositis (DM) is a rare autoimmune inflammatory myopathy characterized by both cutaneous and muscular involvement, often associated with specific autoantibodies and systemic manifestations. This case report presents a young male patient in the United Kingdom with DM, who had a negative myositis blot, panel of autoantibodies, and also had features of myasthenia gravis (MG). Despite trials of multiple immunosuppressive therapies, severe dysphagia persisted. This case underscores the diagnostic complexity of autoimmune neuromuscular disorders and the importance of early consideration of overlap syndromes when clinical features deviate from the classical DM phenotype and bulbar involvement is present. In a healthcare setting where patients may present with diverse and complex autoimmune profiles, this case highlights the need for vigilance and early recognition of rare overlaps such as DM and MG, which is crucial for optimizing patient care.

## Introduction

Dermatomyositis (DM) is a rare idiopathic inflammatory myopathy characterized by symmetric proximal muscle weakness, distinctive cutaneous manifestations, and systemic involvement [1-3], often accompanied by myositis-specific autoantibodies (MSAs) that guide diagnosis and prognosis. The most common of these MSAs are anti-Mi-2 and anti-TIF1-γ, contributing to disease progression and symptomatology. However, up to 20% of patients are seronegative and pose a diagnostic challenge [4].

Myasthenia gravis (MG), in contrast, is an autoimmune disorder of neuromuscular transmission, marked by fatigable weakness of ocular, bulbar, and limb muscles, typically associated with acetylcholine receptor antibodies [5,6]. While the co-occurrence of DM and MG is rare, with fewer than 26 cases reported [6], it has primarily been described in isolated case reports and small case series [5]. Recognition of such overlap syndromes is essential for timely diagnosis and tailored management, particularly in seronegative patients.

Characteristic manifestations of both DM and MG include symptoms such as dysphagia and dysarthria, along with proximal muscle weakness. Bulbar symptoms occur in fewer than 40% of patients with DM and in approximately 70% of those with MG [7].

Here, we present an unusual case of seronegative DM overlapping with MG in a 17-year-old male with persistent dysphagia, highlighting the diagnostic complexity, therapeutic strategies, and clinical significance of such autoimmune overlaps.

## Case presentation

A 17-year-old white British male was admitted to the acute medical department on January 1, 2025, with a three-week history of progressive dysphagia and a three-month history of rash, weight loss, and progressive weakness, culminating in significant disability and inability to perform basic activities. Before symptom onset, he had been a fit, athletic college student with no past medical history.

Neurological examination revealed symmetrical proximal muscle weakness (power 2/5) in both upper and lower limbs, with marked fatigability and associated muscle aches but no fasciculations or wasting. Gait was slow and bilaterally unsteady, with an inability to sustain heel-to-toe walking, limited by proximal weakness. Cranial nerve examination showed fatigable facial weakness, mild ptosis, and fatigue on neck flexion was noted. Swallowing was uncoordinated, with significant dysphagia to semi-solid foods. The ice-pack test was unremarkable. No sensory deficits, coordination issues, or cerebellar signs were noted; based on these features, differentials included peripheral neuropathy, autoimmune myopathy, and myasthenia gravis. A rash involving the upper eyelids, collar region, and knuckles consistent with classical features of dermatomyositis was seen. Laboratory analysis on January 6, 2025, showed a mildly elevated creatine kinase level of 618 U/L (reference ranges for males: European <200 U/L, Afro-Caribbean <600 U/L, Asian <330 U/L). The forced vital capacity (FVC) was 3.98 L, with a predicted value of 5.73 L and a Z score of -2.45. Treatment was initiated on January 7, 2025, with three pulses of intravenous methylprednisolone (500 mg daily for three days). On January 10, 2025, oral prednisolone was started at 50 mg daily, tapered by 10 mg every two weeks to a maintenance dose of 10 mg. Methotrexate was given at 10 mg once weekly for two weeks, then increased to 15 mg once weekly, together with folic acid 5 mg once weekly. Initial treatment resulted in a slight improvement in mobility but no improvement in dysphagia.

**Figure 1:**
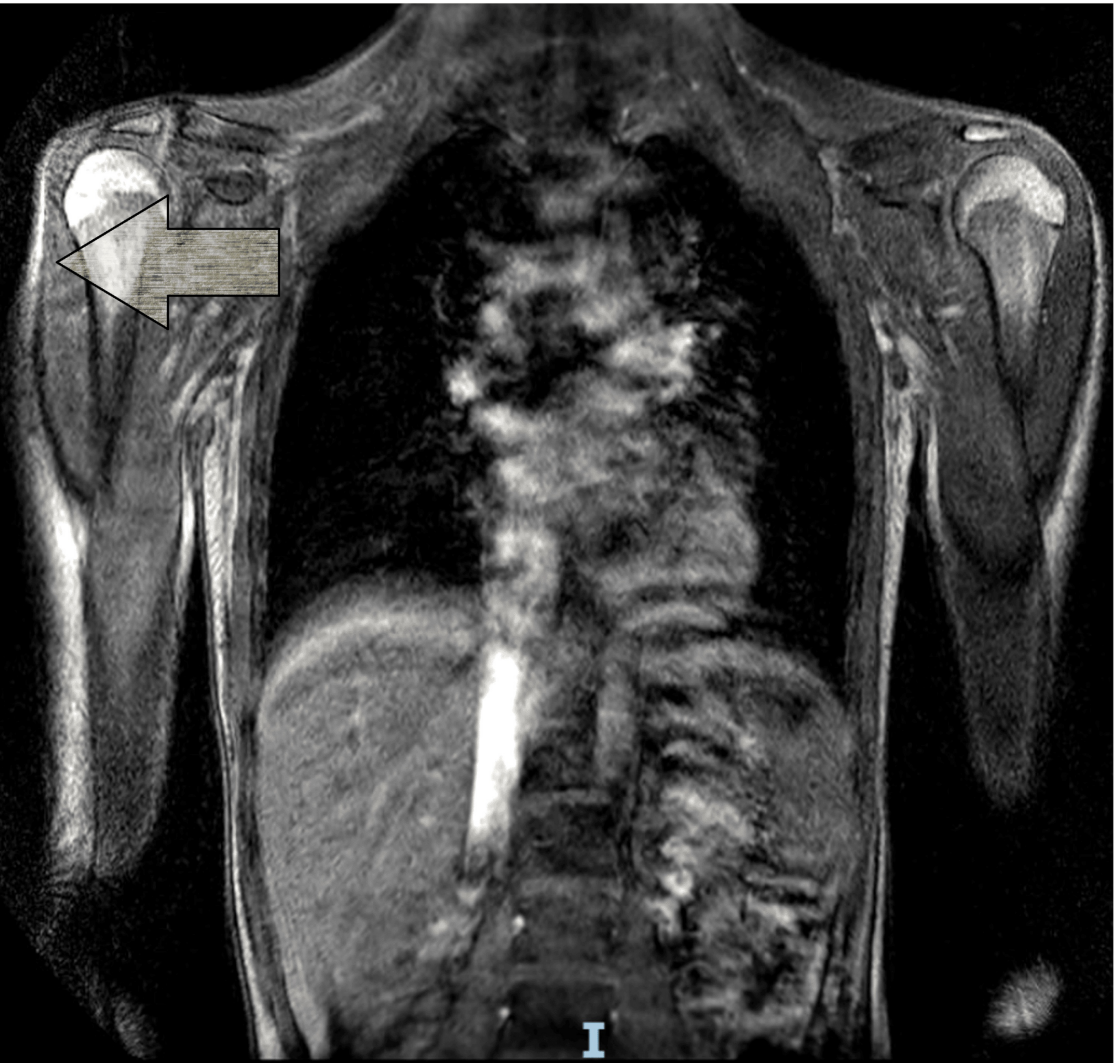
MRI scan of the thorax showing extensive dermatomyositis affecting the shoulder girdle and chest. Features of dermatomyositis are indicated by an arrow. MRI, magnetic resonance imaging

**Figure 2:**
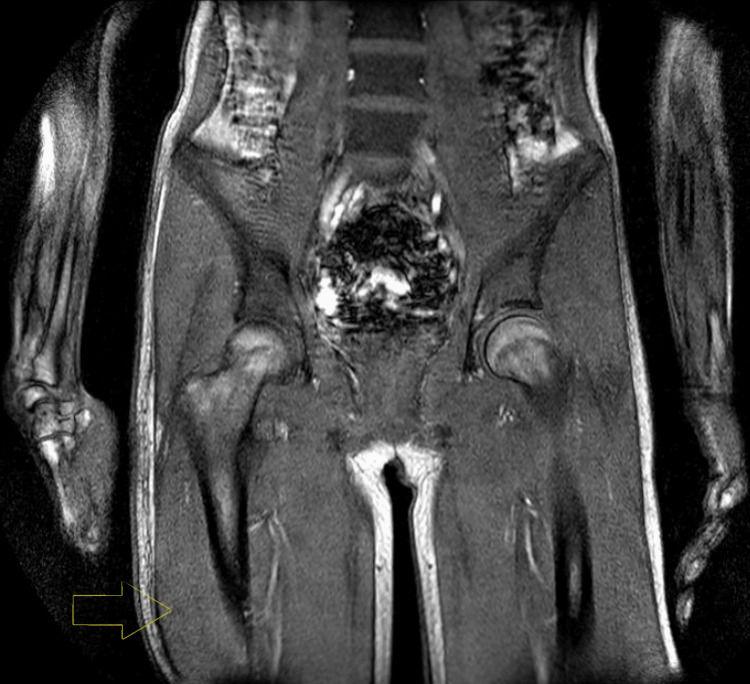
MRI of the lower body showing extensive bilateral involvement of the thigh musculature. Features of dermatomyositis are indicated by an arrow. MRI, magnetic resonance imaging

These clinical features prompted further investigations, including a whole-body MRI performed on January 10, 2025 (Figures 1-2), which demonstrated inflammatory myopathy, while results of myositis-specific autoantibodies (MSAs) were pending.

Although there was visible improvement in the proximal muscles and rash, persistent dysphagia led to the initiation of intravenous immunoglobulin (IVIG) therapy on January 21, 2025. He received three courses of IVIG (160 g each) administered every four to six weeks. Each course was divided over five days: Day 1, 30 g; Day 2, 30 g; Day 3, 30 g; Day 4, 30 g; Day 5, 40 g. After finishing his first course of IVIG, a marked improvement in FVC and dysphagia was noted in one month, although he still required a nasogastric tube for nutritional support. The second course of IVIG was administered on February 15, consisting of 130 g divided over five days (25 g daily for four days and 30 g on the fifth day).

**Figure 3:**
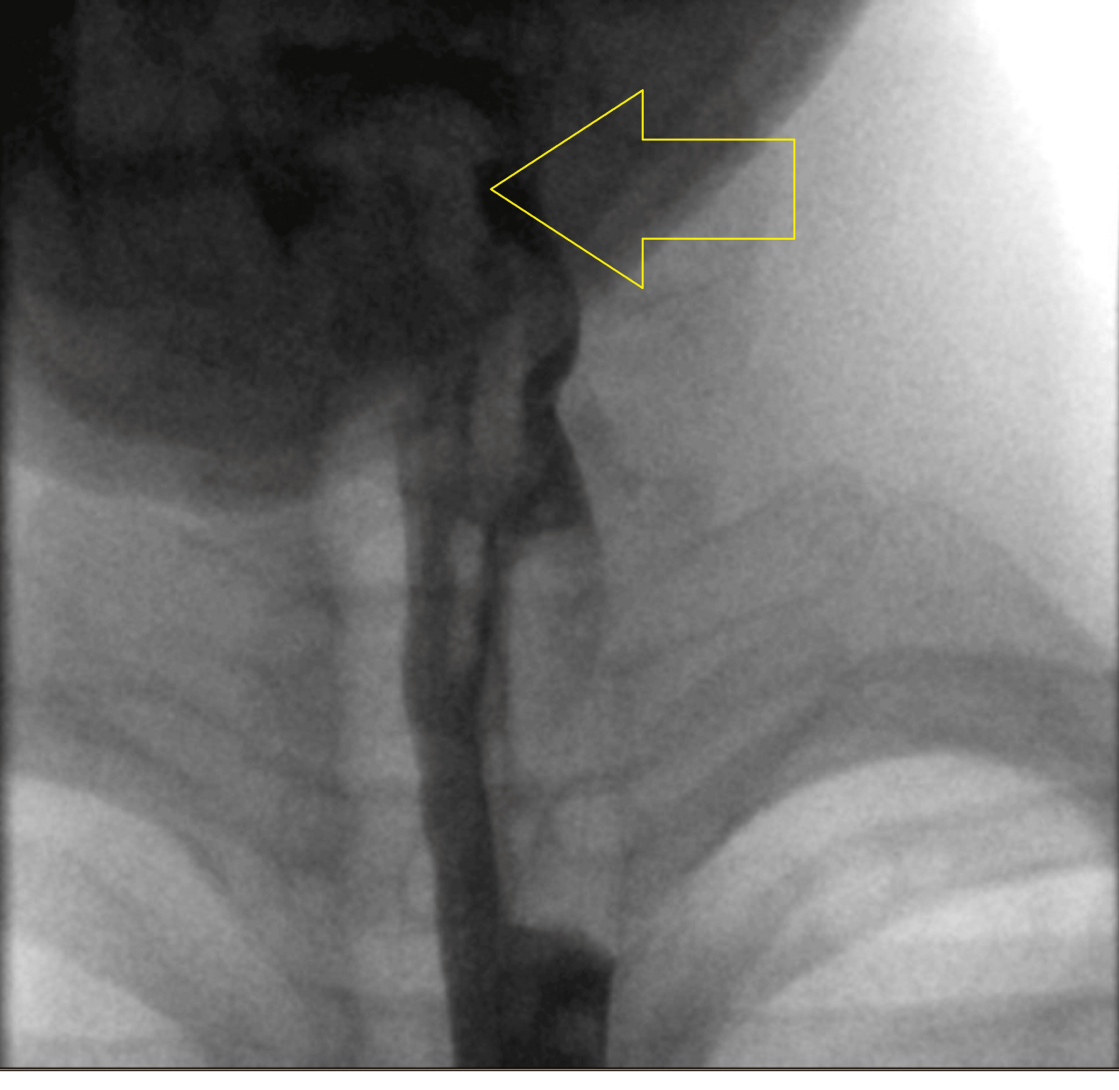
Barium swallow with nasogastric tube in situ showing cricopharyngeal spasm, poor clearance of contrast in the pyriform sinuses bilaterally, and aspiration of contrast below the vocal cords. The extent of dysphagia is shown by the yellow arrow.

Further investigations for persistent dysphagia despite treatment led to a barium swallow, which demonstrated cricopharyngeal spasm, as shown in Figure 3. This led to a trial of baclofen, which resulted in slight improvement, and an esophageal biopsy was essentially normal.

The MSAs, sent on January 10, 2025, were all negative, as shown in Table 1. Other antibody tests, including 3-hydroxy-3-methylglutaryl-CoA reductase (HMGCR) antibodies, were also negative. A muscle biopsy performed on March 5, 2025, demonstrated findings consistent with inflammatory myopathy. Nerve conduction study results were consistent with inflammatory myopathy. Meanwhile, acetylcholine receptor antibodies (AChR Ab) were positive with elevated titers of 1.21 nmol/L (normal range 0.00-0.39 nmol/L). Acetylcholine receptor cluster antibodies, anti-muscle-specific kinase (anti-MuSK) antibodies, and low-density lipoprotein receptor-related protein 4 (LRP4) antibodies were negative, as shown in Table 2.

**Table 1:** Results of myositis-specific antibodies testing.

Myositis blot antibodies	Result
Anti-Mi-2 alpha (microsomal antigen Mi-2 alpha)	Negative
Anti-Mi-2 beta (Microsomal Antigen Mi-2 beta)	Negative
Anti-TIF1 gamma (transcription intermediary factor 1 gamma)	Negative
Anti-MDA5 (melanoma differentiation-associated gene 5)	Negative
Anti-NXP2 (nuclear matrix protein 2)	Negative
Anti-SAE1 (small ubiquitin-like modifier (SUMO) activating enzyme subunit 1)	Negative
Anti-Ku (Ku antigen)	Negative
Anti-PM-SCL-100 (polymyositis–scleroderma antigen, 100 kDa subunit)	Negative
Anti-PM-SCL-75 (polymyositis–scleroderma antigen, 75 kDa subunit)	Negative
Anti-Jo-1 (histidyl-tRNA synthetase)	Negative
Anti-SRP (signal recognition particle)	Negative
Anti-PL-7 (threonyl-tRNA synthetase)	Negative
Anti-PL-12 (alanyl-tRNA synthetase)	Negative
Anti-EJ (glycyl-tRNA synthetase)	Negative
Anti-OJ (isoleucyl-tRNA synthetase)	Negative
Anti-Ro 52 (52 kDa Ro/SSA protein)	Negative

**Table 2:** Results of AChR clustered antibodies. MuSK, muscle-specific kinase; LRP4, low-density lipoprotein receptor-related protein 4

Acetylcholine receptor (AChR) clustered antibodies	Result
Anti-AChR cluster	Negative
Anti-MuSK	Negative
Anti-LRP4	Negative

With no further improvement after prolonged treatment, on February 6, 2025, he was started on cyclosporine 50 mg once daily, in addition to methotrexate and a trial of pyridostigmine 30 mg four times daily. The following day, a significant improvement in mobility was noted, and the dose of pyridostigmine was doubled.

He started intensive physiotherapy sessions, and over the next few weeks, we noted improvements in swallowing and mobility. He was discharged on March 14, 2025, with a nasogastric tube and the following medications: baclofen 10 mg three times daily, prednisolone 12.5 mg once daily, pyridostigmine 75 mg four times daily, cyclosporine 100 mg twice daily, folic acid 5 mg once weekly, and methotrexate 15 mg once weekly. 

He was readmitted on June 4, 2025, with muscle weakness, generalized body aches, and dysphagia. A repeat whole-body MRI demonstrated diffuse myositis, similar to the previous study. He was started on a higher dose of prednisolone and cyclosporine, and a third course of IVIG was administered. A repeat muscle biopsy was performed, revealing prominent perifascicular fiber atrophy with minimal inflammation on paraffin sections, consistent with a diagnosis of inflammatory dermatomyositis. His symptoms improved, and he was discharged on a tapering dose of medication.

## Discussion

This case illustrates an unusual autoimmune overlap syndrome involving dermatomyositis and myasthenia gravis in a young male. The patient presented with symptoms suggestive of both conditions; however, the absence of detectable myositis-specific antibodies complicated the diagnostic process.

The coexistence of DM and MG is rare, with fewer than 50 cases reported to date [5,7]. In most instances, MG either precedes or follows the diagnosis of DM, making simultaneous presentation, as in this case, uncommon. The seronegative nature of the patient’s DM is consistent with findings in ~20% of cases, where diagnosis relies on clinical, histological, and imaging features [7].

The positive acetylcholine receptor antibodies and the patient’s response to pyridostigmine supported the diagnosis of MG. Meanwhile, muscle biopsy findings of perifascicular atrophy and inflammation were characteristic of DM. Notably, cricopharyngeal spasm and hypercontractile esophageal contractions have been observed in other cases of seronegative DM [7].

Initial standard immunosuppressive therapy had a limited effect, highlighting the need for individualized, multimodal management in overlap syndromes. The patient improved following IVIG, acetylcholinesterase inhibition, and supportive treatments, consistent with existing case reports advocating multidisciplinary approaches [5,7].

This case reinforces the importance of maintaining a high index of suspicion for overlap syndromes in atypical or treatment-resistant presentations. It also highlights the limitations of antibody panels and the indispensable role of muscle biopsy, EMG, and MRI in clarifying the diagnosis.

## Conclusions

This case highlights the complexities of diagnosing and treating autoimmune overlap syndromes, particularly when standard serological markers are absent. In young patients presenting with muscle weakness, rash, and fatigue, clinicians should consider both DM and MG, even when antibody tests are negative. Early identification and a multidisciplinary treatment approach can improve outcomes in such rare presentations. Further research is needed to identify novel biomarkers and optimize treatment protocols for seronegative and overlapping autoimmune myopathies.
